# The Adsorption of Cd(II) on Manganese Oxide Investigated by Batch and Modeling Techniques

**DOI:** 10.3390/ijerph14101145

**Published:** 2017-09-28

**Authors:** Xiaoming Huang, Tianhu Chen, Xuehua Zou, Mulan Zhu, Dong Chen, Min Pan

**Affiliations:** 1 Laboratory of Nano-Minerals and Environmental Materials, School of Resources and Environmental Engineering, Hefei University of Technology, Hefei 230009, China; huangxm@xmut.edu.cn (X.H.); zouxuehua@mail.hfut.edu.cn (X.Z.); cdxman@hfut.edu.cn (D.C.); 2 School of Environmental Science and Engineering, Xiamen University of Technology, Xiamen 361024, China; zhuml@xmut.edu.cn

**Keywords:** cadmium, Mn oxides, adsorption, surface complexation modeling

## Abstract

Manganese (Mn) oxide is a ubiquitous metal oxide in sub-environments. The adsorption of Cd(II) on Mn oxide as function of adsorption time, pH, ionic strength, temperature, and initial Cd(II) concentration was investigated by batch techniques. The adsorption kinetics showed that the adsorption of Cd(II) on Mn oxide can be satisfactorily simulated by pseudo-second-order kinetic model with high correlation coefficients (R^2^ > 0.999). The adsorption of Cd(II) on Mn oxide significantly decreased with increasing ionic strength at pH < 5.0, whereas Cd(II) adsorption was independent of ionic strength at pH > 6.0, which indicated that outer-sphere and inner-sphere surface complexation dominated the adsorption of Cd(II) on Mn oxide at pH < 5.0 and pH > 6.0, respectively. The maximum adsorption capacity of Mn oxide for Cd(II) calculated from Langmuir model was 104.17 mg/g at pH 6.0 and 298 K. The thermodynamic parameters showed that the adsorption of Cd(II) on Mn oxide was an endothermic and spontaneous process. According to the results of surface complexation modeling, the adsorption of Cd(II) on Mn oxide can be satisfactorily simulated by ion exchange sites (X_2_Cd) at low pH and inner-sphere surface complexation sites (SOCd^+^ and (SO)_2_CdOH^−^ species) at high pH conditions. The finding presented herein plays an important role in understanding the fate and transport of heavy metals at the water–mineral interface.

## 1. Introduction

The contamination of heavy metals is of great concern to humans due to their toxicity, bioaccumulation, and non-biodegradation [1]. Cadmium (Cd) is a non-essential and highly toxic heavy metal to all living organisms including animals, plants, and human beings [2]. The World Health Organization recommended the drinking water guideline value to be 0.005 mg Cd/L [3]. Therefore, it is compulsory to remove Cd(II) from water before its transport and cycling into the natural environment. The removal of Cd(II) and other heavy metals from water and wastewater has been recently investigated by using various methods such as adsorption, chemical precipitation, electrodialysis, ion exchange, membrane separation, and redox [4,5]. Among these methods, adsorption is considered as an effective technique due to its properties of simply operation, low cost, and high efficiency over a wide concentration range of pollutant [6]. Typical adsorbents—including clay minerals [7,8], carbon-based materials [9,10,11], and metal oxides [12,13]—have been extensively employed to remove heavy metals. Great attention has been paid to research on different types of low-cost natural and modified minerals for the removal of Cd(II) from aqueous solutions.

Owing to the high surface charge density, various Mn oxides (e.g., including pyrolusite (β-MnO_2_) and birnessite (δ-MnO_2_)) have been extensively as high efficient adsorbents to remove arsenic [14,15,16,17], nickel [18,19], lead [20,21,22], mercury [23,24], and chromium [25,26,27]. It is demonstrated that birnessite has hexagonal layer symmetry and may possess a variable number of octahedral cationic vacancies within their layer, which provides the abundant strong adsorption sites for heavy metals [28,29]. The fate and transport of Cd(II) at water-mineral interface is affected greatly by solution chemistry such as pH, ionic strength and metal speciation [30]. Although there are abundant studies on the adsorption of Cd(II) on Mn oxide, there is little information on interaction mechanism using surface complexation modeling available [31,32,33].

The objectives of this study were to synthesize Mn oxide and characterize it by using X-ray diffraction (XRD) and specific surface area analysis. The effects of various environmental conditions such as adsorption time, pH, ionic strength, and initial Cd(II) concentration on Cd(II) adsorption on Mn oxide were investigated by batch experiments. The adsorption isotherms, thermodynamics, and kinetics were applied to discuss the adsorption mechanism. The interaction mechanism was determined by using surface complexation modeling. The highlight of this study is the practical application of Mn oxide for the preconcentration and immobilization of heavy metals in environmental cleanup.

## 2. Materials and Methods

### 2.1. Materials

Mn oxides were synthesized by reducing KMnO_4_ with a stoichiometric amount of MnSO_4_ solution [34]. Briefly, a solution of 13.5 g MnSO_4_·H_2_O and 3.95 g MgSO_4_·7H_2_O in 160 mL deionized water was added slowly to 180 mL of 5.5 M NaOH with magnetic stirring. Pink gels of Mn(OH)_2_ were formed in the beaker. Then 160 mL solution with 5.1 g KMnO_4_ was added slowly into the suspension under vigorously stirring condition. This black suspension was aged at room temperature for 60 days. The resultant precipitate was washed with deionized water and centrifuged repeatedly until conductivity was less than 10 μs/cm.

The stock Cd(II) solution (1.0 g/L) was prepared by dissolving Cd(NO_3_)_2_·4H_2_O (99.99% purity grade, Sigma-Aldrich, St. Louis, MO, USA). Different concentrations of Cd(II) solutions were obtained by diluting with the stock solution. All other reagents of analytical grade were purchased from Sinopharm Chemical Reagent Co., Ltd., Shanghai, China, and were used directly without further purification.

### 2.2. Batch Adsorption

The batch adsorption experiments were conducted at pH 6 in the presence of 0.01 mol/L NaClO_4_ conditions. The pH was adjusted to 6 in order to avoid the possible formation of Cd(OH)_2_ (s) precipitates. The adsorption kinetics was performed at pH 6 and 0.01 mol/L NaClO_4_ under the different adsorption time of 15, 30, 60, 90, 120, 180, and 240 min. For the kinetic test, the initial solution volume was 1000 mL and 2.0 mL aliquots were sampled at various time intervals. The influence of pH and ionic strength on Cd(II) adsorption was investigated as the previous procedure except that the solution pH was adjusted to the desired values under the different ionic strength conditions (0.001, 0.01, and 0.1 mol/L NaClO_4_ solution). The adsorption isotherms were studied with the same procedure as described above (pH = 6, I = 0.01 mol/L NaClO_4_, solution was shaken for 24 h), except that the initial Cd(II) concentration was varied from 1.0 to 100 mg/L at different temperature (298, 308, and 318 K). The blank experiments also showed that Cd(II) adsorption to glass slides was negligible. After adsorption, the solid-phase was separated from liquid-phase by centrifuging it at 4000 rpm for 15 min and then was filtered through a 0.45 μm nylon membrane. The concentration of Cd(II) in aqueous solutions was measured by flame atomic absorption spectrometry (AA-6300C, Shimadzu, Kyoto, Japan). The adsorptive capacity and the adsorption efficiency (R) can be obtained by Equations (1) and (2)

*q_t_* = (*C*_0_ − *C_t_*) × *V*/*m* (1)

R = (*C*_0_ − *C_e_*) × 100%/*C*_0_ (2)

where *q_t_* (mg/g) is the adsorptive capacity at time *t*; *C*_0_ and *C_e_*(mg/L) are the initial and equilibrium Cd(II) concentrations, respectively; *C_t_* (mg/L) is the concentration of Cd(II) at time *t*; *V* (L) is the volume of the solution; and *m* (g) is the mass of adsorbent.

### 2.3. Characterization

Mineral phases were identified by XRD using a D/max-RB powder diffraction meter (Rigaku, Tokyo, Japan), with a Cu-target operated at 40 kV, 100 mA. The XRD pattern was taken in the range of 5–70° at a scan speed of 4°/min, which was analyzed using the software (Search-Match, Almelo, The Netherlands) by comparing the experimental data with those included in the Joint Committee of Powder Diffraction Standards (JCPDSs) database. The multi-point BET surface area of the sample was measured at atmospheric pressure using Quantachrome NOVA 3000e surface area and pore size analyzer (Quantachrome Instruments, Bointon Beach, FL, USA).

### 2.4. Surface Complexation Modeling

The distribution of Cd speciation in aqueous solutions at [Cd] = 1.0 × 10^−6^ mol/L was calculated using Visual MINTEQ. According to the survey of the previous studies [35,36], the cation exchange and surface complexation sites were employed to simulate the adsorption of Cd(II) on Mn oxide. The reactions of cation exchange and surface complexation can be expressed as Equations (3)–(5)

2XH + Cd^2+^ = X_2_Cd + 2 H^+^ (3)

SOH + Cd^2+^ = SOCd^+^ + H^+^ (4)

2SOH + Cd^2+^ + H_2_O = (SO)_2_CdOH^−^ +3 H^+^ (5)

where XH and SOH refer to the cation exchange and surface complexation sites, respectively. These equilibrium constants (log *K* values) can be optimized the fitted results compatible with the experimental data so far as possible.

## 3. Results and Discussion

### 3.1. Characterization

**Figure 1:**
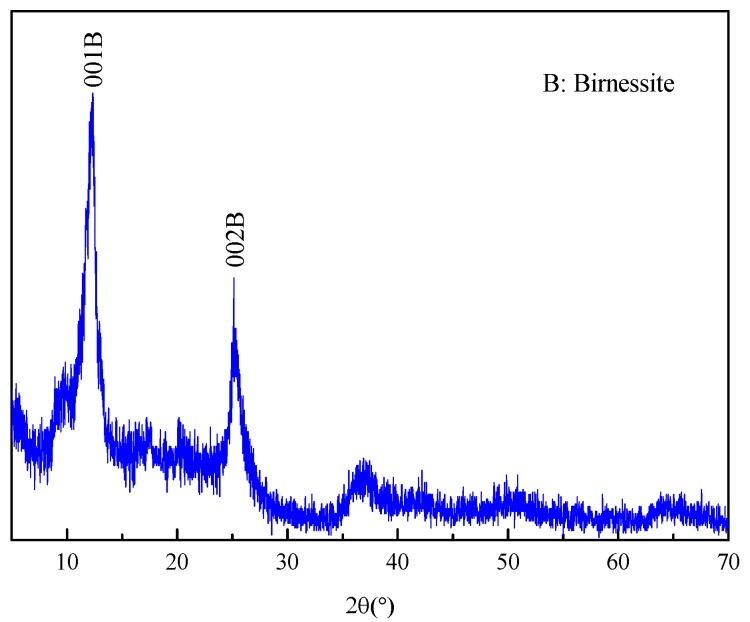
The XRD pattern of Mn oxide.

According to XRD analysis from Figure 1, the feature peaks at 2θ angles of 12.36° and 25.14° were assigned to (110) and (002) plane, respectively, which was identified as birnessite [34]. The specific surface area of Mn oxide was calculated to be 84.9 m^2^/g according to BET method. The average pore size and microvolume of Mn oxide were 11.32 nm and 0.074 cm^3^, respectively.

### 3.2. Adsorption Kinetics

**Figure 2:**
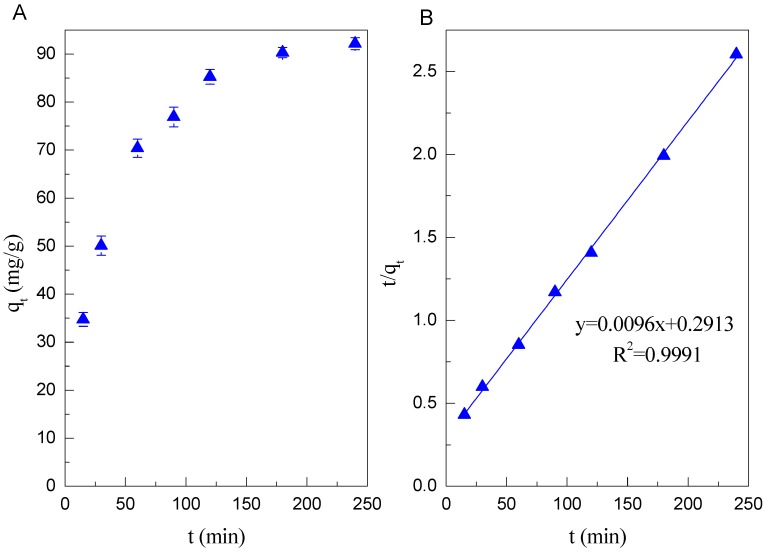
(**A**) The adsorption kinetics of Cd(II) on Mn oxide (pH = 6, *C* = 100 mg/L, *I* = 0.01 mol/L NaClO_4_, *m*/*v* = 1 g/L, *T* = 298 K); (**B**) Pseudo second-order kinetic plot.

The adsorption kinetics of Cd(II) on Mn oxide is conducted by batch technique. It is shown from Figure 2A that the adsorption of Cd(II) on Mn oxide is very fast within 3 h. More than 90% of Cd(II) is removed after 3 h. Such a fast adsorption could be ascribed to the large surface area and mesoporous structures. The data of adsorption kinetics were fitted by pseudo-first-order and pseudo-second-order models. Their linear equations can be described by Equations (6) and (7)

(6) $$\mathrm{Pseudo}\;\mathrm{first-order}\;\mathrm{equation}:\;\ln(q_{e}-q_{t})=\ln q_{e}-k_{1}t$$

(7) $$\mathrm{Pseudo}\;\mathrm{second-order}\;\mathrm{equation}:\;\frac{t}{q_{t}}=\frac{1}{k_{2}q_{e}^{2}}+\frac{t}{q_{e}}$$

where *q_t_* and *q_e_* are the amount of adsorbed adsorbate (mg/g) on adsorbent at equilibrium and time *t*, respectively. *k*_1_ (min^−1^) and *k*_2_ (g/(mg·min)) are the rate constants for pseudo-first-order and pseudo-second-order adsorption, respectively.

As shown in the Figure 2B, the adsorption kinetics of Cd(II) on Mn oxide can be fitted very well to a pseudo-second-order kinetic model with the high correlation coefficients (R^2^ > 0.999) (Table 1).

### 3.3. Effect of pH and Ionic Strength

**Figure 3:**
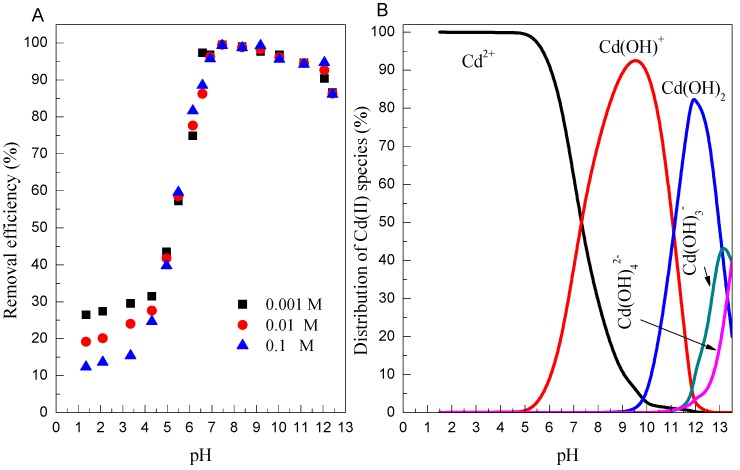
(**A**) Effect of pH on Cd(II) adsorption onto Mn oxide (*C* = 10 mg/L, *m*/*v* = 1 g/L, *T* = 298 K); (**B**) The distribution of Cd(II) species in aqueous solutions (*I* = 0.01 mol/L NaClO_4_, *T* = 298 K).

The effect of the initial solution pH on Cd(II) adsorption by Mn oxide is showed in Figure 2, with the pH value ranging between 1.0 and 13.0. The adsorption percentage of Cd(II) on Mn oxide is little influenced at pH < 4.0, whereas significantly increases upon increasing the pH from 4.0 to 6.0, and it then remains constant (approximately 98%) in the pH range of 6.0–9.0. However, it reduces to 85% at pH 13.0. The dependence of Cd(II) adsorption on pH could be explained by the relative distribution of Cd(II) species in aqueous solution and the surface chemistry of Mn oxide [37,38]. As shown in Figure 3B, the main Cd(II) species is Cd^2+^, Cd(OH)^+^, and Cd(OH)_2_ species at pH < 7.0, 7.0–11.0, and 11.0–13.0, respectively. As measured in the previous studies [39], Mn oxide is negatively charged above pH 3.5, thus the enhanced adsorption of Cd(II) on Mn oxide in the pH range of 4.0–11.0 is mainly governed by electrostatic attractions between negative charged of Mn oxide and positive charged Cd(II) species such as Cd^2+^ and Cd(OH)^+^ species [40]. However, the decreased adsorption of Cd(II) at pH > 11.0 could be due to the weak electrostatic attraction because Cd(OH)_2_ (aq) species was difficult to be adsorbed on the Mn oxide surface. The pH-dependent adsorption of Cd(II) on Mn oxide could be reasonably explained by the ion-exchange process at low pH conditions.

The common ions present in wastewater may compete with the heavy metal ions for the available binding sites of adsorbents, affecting the adsorption process to some degree. Figure 3A also presents the adsorption of Cd(II) at various NaClO_4_ concentrations. It is observed that the adsorption of Cd(II) on Mn oxide significantly decreases as the ionic strength increased from 0.001 to 0.1 mol/L between pH 1 to 4, whereas no significant effect of ionic strength on Cd(II) adsorption on Mn oxide is observed at pH ≥ 5.0. The inhibited effect of ionic strength can be interpreted by (i) the electrolyte ion (Na^+^) compete with positively charged heavy metal ions from the same binding sites; and (ii) the ionic strength influences the interfacial potential of heavy metals, which would in turn limit their transfer to the adsorbent surface [40]. The interaction mechanism of Cd(II) adsorption onto Mn oxide could be explored in terms of ionic strength dependence results, which can provide molecular evidence for the formation of inner- vs. outer-sphere surface complexes. It has been demonstrated that the outer-sphere complexation is more sensitive to ionic strength, whereas inner-sphere surface complexation is independent of ionic strength [41]. The experimental data suggests that the outer-sphere surface complexation dominates the adsorption of Cd(II) on Mn oxide at pH < 5.0, whereas adsorption of Cd(II) on Mn oxide at pH > 6.0 is inner-sphere surface complexation.

### 3.4. Adsorption Isotherms

**Figure 4:**
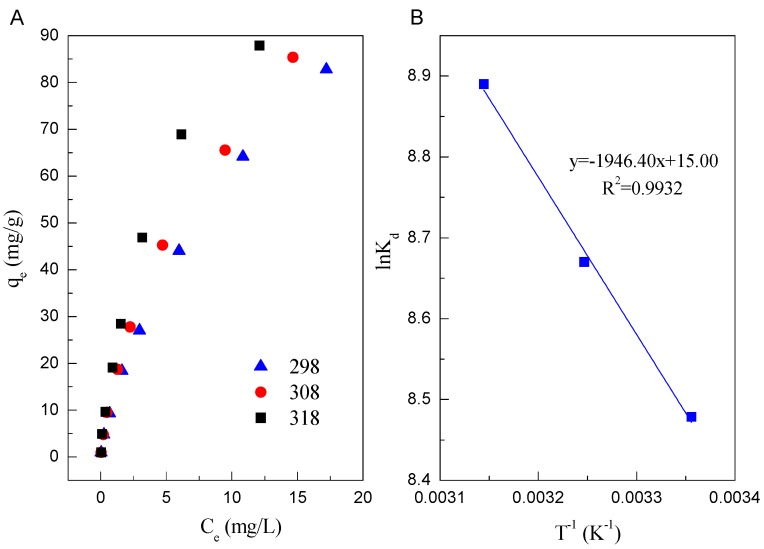
(**A**) Adsorption isotherms of Cd(II) on Mn oxide (pH = 6.0, *C* = 1, 5, 10, 20, 30, 50, 75, 100 mg/L, *I* = 0.01 mol/L NaClO_4_, *m/v* = 1 g/L, *T* = 298 K); (**B**) Plot of ln*K_d_* vs. 1/*T.*

Figure 4A shows the adsorption isotherms of Cd(II) on Mn oxide. The highest and lowest adsorption of Cd(II) on Mn oxide were observed at *T* = 318 K and 298 K, respectively, indicating enhanced adsorption of Cd(II) on Mn oxide at high temperature. The data of adsorption isotherms were fitted by Langmuir and Freundlich model. The Langmuir model is based on the assumption that adsorption occurs at single specific sites [42], whereas Freundlich model assumes that the strong binding sites are superior and the binding affinity decreases with an increasing degree of site occupation [43]. Their linear equations can be expressed by Equations (8) and (9)

(8) $$\frac{C_{e}}{q_{e}}=\frac{1}{q_{m}}C_{e}+\frac{1}{q_{m}k}$$

(9) $$\ln q_{e}=\ln K_{f}+\frac{1}{n}\ln C_{e}$$

where *q_e_* (mg/g) and *q_m_* (mg/g) are the equilibrium and maximum adsorption capacity corresponding to complete monolayer coverage, respectively. *K_f_* (mg/g) and *k* (L/mg) are the Freundlich and Langmuir constants, respectively.

The fitted results of two isotherm models are listed in Table 2. Base on the correlation coefficient values, it can be seen that the adsorption of Cd(II) on Mn oxide can be fitted by Freundlich model compared to Langmuir model. The fitted results indicate that the adsorption occurs on a structurally heterogeneous adsorbent [44]. The maximum adsorption capacity (*q_m_*) on monomolecular coverage of Mn oxide toward Cd(II) calculated from Langmuir model is 104.17 mg/g at pH 6.0 and 298 K. The high adsorption of Cd(II) on Mn oxide could be attributed to the ion exchange [45].

### 3.5. Thermodynamic Parameters

The thermodynamic parameters (Gibbs free energy change—Δ*G*^0^, standard enthalpy change—Δ*H^0^* standard entropy change—Δ*S*^0^) of the adsorption of Cd(II) on Mn oxide can be determined from the temperature-dependent uptake data. The value of Δ*G*^0^ can be calculated by Equation (10)

(10) $$\Delta G^{0}=-RT\ln K_{d}$$

where *R* and *T* are the universal constant (8.314 J/(mol·K) and the temperature (K), respectively. *K_d_* (L/g) is the adsorption equilibrium constant and can be obtained by dividing *q_e_* by *C_e_*. The values of Δ*H*^0^ and Δ*S*^0^ can be calculated from the slope and intercept of plot of ln*K_d_* versus 1/*T* according to Equation (11)

(11) $$\ln K_{d}=-\frac{\Delta H^{0}}{RT}+\frac{\Delta S^{0}}{R}$$

The calculation of theses thermodynamic parameters are tabulated in Table 3. As shown in Table 3, the values of Δ*G*^0^ at different temperatures are negative as expected for a spontaneous process under the experimental conditions. The values of Δ*G*^0^ (−21.006 kJ/mol at 298 K and −23.503 kJ/mol at 318 K, respectively) become more negative with increasing temperature, which show that the adsorption process is more favorable at higher temperature due to the dehydration of Cd(II) ions. A positive value of Δ*H*^0^ (16.182 kJ/mol) indicates that the adsorption of Cd(II) on Mn oxide is an endothermic process. The positive value of Δ*S*^0^ (0.125kJ/(mol·K)) reveals some structural changes in Cd(II) ions and Mn oxide during the sorption process, which lead to an increase in the disorder of the solid-solution system during the adsorption of Cd(II) on Mn oxide. Besides, the entropy of activation (Δ*S*^0^) parameter is generally regarded as a measure of the saddle point width of the potential energy surface over which reactant molecules must pass to act as activated complexes.

### 3.6. Effect of Coexisting Cations

Alkaline earth cations such as Ca^2+^ and Mg^2+^ are ubiquitous in the environment that may compete with heavy metals for the binding sites of adsorbents. Therefore, the influence of coexisting cations on adsorption of Cd(II) was investigated in 1 mmol/L and 10 mmol/L Ca(NO_3_)_2_ and Mg(NO_3_)_2_ solutions, respectively. The results listed in Table 4 indicate that the adsorption capacity of Cd(II) by Mn oxide was slightly influenced by high level coexisting cations. It can be attributed to covalent bonds between the adsorbed metal ions and the surface functional groups in an inner-sphere surface complex [36]. It has been reported that adsorption of Cd(II) by Mn oxide may be attributed to formation of inner-sphere surface complexes due to their insensitivity to Ca^2+^ and Mg^2+^ [46]. These results proved that Mn oxides are a potential preferable absorbent for heavy metals removal. This view is consistent with the conclusion from the influence of ionic strength on the adsorption of Cd(II).

### 3.7. Surface Complexation Modeling

**Figure 5:**
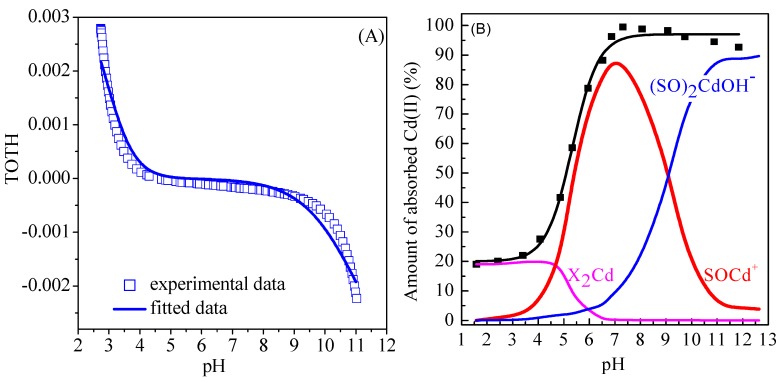
(**A**) Fitting of potentiometric data of Cd(II) on Mn oxide in the presence of 0.01 mol/L NaClO_4_; (**B**) Surface complexation modeling of Cd(II) on Mn oxide (*C* = 10 mg/L, *I* = 0.01 mol/L NaClO_4_, *m/v* = 1 g/L, *T* = 298 K).

The pH-dependent adsorption of Cd(II) on Mn oxides was fitted by surface complexation modeling using Visual MINTEQUATION MINTEQ model contains subroutines for computing the surface complexation with a nonelectrostatic model [47]. The constants of protonation and deprotonation (log *K*^+^ and log *K*^−^ values) were calculated by fitting the potentiometric titration data. Briefly, the reactions of protonation and deprotonation can be described as Equations (12) and (13), respectively

(12) $$\mathrm{SOH}+H^{+}=SO{H_{2}}^{+}\;\log K^{+}=\frac{\log[SO{H_{2}}^{+}]}{\log([SOH]\times [H^{+}])}$$

(13) $$\mathrm{SOH}=SO^{-}+H^{+}\;\log K^{-}=\frac{\log([SO^{-}]\times [H^{+}])}{\log[SOH]}$$

where SOH refers to amphoteric surface reactive sites of Mn oxides, SOH_2_^+^ and SO^−^ refer to the protonation and deprotonation ions, respectively. The values of log *K*^+^ and log *K*^−^ were obtained by fitting the potentiometric titration data using Visual MINTEQ software. Figure 5A shows the fitting of potentiometric titration of Mn oxide in the presence of 0.01 mol/L NaClO_4_ solution. The fitted results of surface complexation modeling and the corresponding parameters are showed in Figure 5B and Table 5, respectively. As shown in Figure 5B, the adsorption of Cd(II) on Mn oxide can be satisfactorily simulated using diffuse layer model with cation exchange sites (XH) and surface complexation sites (SOH). The main adsorption species are X_2_Cd and SOCd^+^ species at pH < 4.0 and pH 4.0–8.0, respectively, whereas the (SO)_2_CdOH^−^ species dominates the adsorption of Cd(II) on Mn oxide at pH > 9.0. The fitted results indicate that the adsorption of Cd(II) on Mn oxide is mainly cation exchange at pH < 4.0, whereas the inner-sphere complexes dominates the adsorption of Cd(II) on Mn oxide at pH > 5.0. In addition, the transformation of inner-sphere complexes from the mononuclear monodentate complexes (SOCd^+^ species) to mononuclear bidentate complexes ((SO)_2_CdOH^−^) is observed with increasing pH in aqueous solutions.

## 4. Conclusions

Mn oxide was successfully prepared by a facile hydrothermal method without any template and organic surfactant. The batch adsorption experiments indicated that Mn oxide could achieve a fast and efficient adsorption of Cd(II) from aqueous solutions. The pseudo-second order model and Freundlich model respectively provided the best description of the adsorption kinetics and isotherm of Cd(II) onto Mn oxide. Thermodynamic parameters revealed that the adsorption of Cd(II) on Mn oxide was an endothermic and spontaneous process. Based on the surface complexation modeling, the adsorption mechanism between Cd(II) and Mn oxide is mainly ion exchange at pH < 4.0, whereas the inner-sphere complexes dominates the adsorption of Cd(II) on Mn oxide at pH > 5.0. Considering the simple fabrication procedure, environmentally friendly, and excellent adsorption performance of Mn oxide, it is expected that Mn oxide has broad applications for the preconcentration and immobilization of heavy metal ions from aqueous solutions in environmental cleanup.

## Figures and Tables

**Table 1:** Kinetic parameters of pseudo-first-order and pseudo-second-order kinetic model for Cd(II) adsorption on Mn oxide.

Metal	Pseudo-First-Order			Pseudo-Second-Order		
	*q_e_* (mg/g)	*k*_1_ (g/(mg·min))	R^2^	*q_e_* (mg/g)	*k*_2_ (g/(mg·min))	R^2^
Cd(II)	80.2	0.0205	0.9941	104.2	0.00032	0.9991

**Table 2:** Relative parameters of Langmuir and Freundlich model for Cd(II) adsorption on Mn oxide.

*T* (K)	Langmuir Model			Freundlich Model		
	*q_m_* (mg/g)	*k* (L/mg)	R^2^	*K_f_* (mg/g)	1/n	R^2^
298 K	104.17	0.1627	0.8889	12.95	0.665	0.9994
308 K	107.53	0.1958	0.9446	14.44	0.714	0.9930
318 K	109.89	0.2826	0.9610	19.28	0.702	0.9898

**Table 3:** Thermodynamic parameters of Cd(II) adsorption on Mn oxide.

*T* (K)	Δ*G*^0^ (kJ/mol)	Δ*S*^0^ (kJ/mol/K)	Δ*H*^0^ (kJ/mol)
298	−21.006		
308	−22.202	0.125	16.182
318	−23.503		

**Table 4:** Influence of cations on the adsorption of Cd(II) on Mn oxide.

Heavy Metal	Coexisting Cations	Adsorption Capacity at Different Concentrations of Coexisting Cations (mg/g)		
		0 mmol/L	1 mmol/L	10 mmol/L
Cd(II)	Ca^2+^	82.79	79.56	81.22
	Mg^2+^		83.13	81.37

Experimental conditions: pH = 6.0, *C* = 100 mg/L, *m/v* = 1 g/L, *T* = 298 K.

**Table 5:** The optimized parameters of surface complexation modeling of Cd(II) on Mn oxide.

Equations	Log *K*
Protonation and deprotonation	
SOH + H^+^ = SOH_2_^+^	5.21
SOH = SO^−^ + H^+^	−8.94
Surface complexation modeling	
2XH + Cd^2+^ = X_2_Cd + 2H^+^	4.64
SOH + Cd^2+^ = SOCd^+^ + H^+^	5.72
2SOH + Cd^2+^ + H_2_O = (SO)_2_CdOH^−^ + 3H^+^	−10.63
